# Seed Heteromorphism and Effects of Light and Abiotic Stress on Germination of a Typical Annual Halophyte *Salsola ferganica* in Cold Desert

**DOI:** 10.3389/fpls.2017.02257

**Published:** 2018-01-17

**Authors:** Yali Ma, Juan Wang, Jinghua Zhang, Shiyue Zhang, Yanxia Liu, Haiyan Lan

**Affiliations:** ^1^College of Resource and Environment Sciences, Xinjiang University, Urumqi, China; ^2^Xinjiang Key Laboratory of Biological Resources and Genetic Engineering, College of Life Science and Technology, Xinjiang University, Urumqi, China; ^3^Xinjiang Education Institute, Urumqi, China; ^4^Institute of Economic Crops, Xinjiang Academy of Agricultural Sciences, Urumqi, China

**Keywords:** dormancy type, germination, light effect, seed heteromorphism, *Salsola ferganica*

## Abstract

Seed heteromorphism is a common characteristic of halophyte and an adaptation to the spatial and temporal variations of natural habitats. Differences in dormancy and germination requirements have been documented in heteromorphic seeds of many species, but the mechanisms for maintenance between different status in various populations have not been well-understood. *Salsola ferganica* is a typical annual halophyte in Chenopodiaceae distributed in cold desert, in the present study, we found that it could produce three distinct types of seed according to the shape and size of winged perianth (WP), which differed in dispersal ability, dormancy and germination behaviors. Our further investigation revealed that light could significantly promote germination of heteromorphic seeds of *S. ferganica*, and WP inhibited while GA_3_ enhanced germination, which suggests that *S. ferganica* seeds possessed a photo-sensitive combined with morphological and non-deep physiological dormancy type, in which light was the dominant factor. Not like other desert plant species, the germinability of heteromorphic seeds of *S. ferganica* could not sustain for long (only 1–2 years), especially the small seeds, and was affected by storage time, temperature, salinity, even the environmental conditions of the maternal plant. Thus, the differences of characteristics existed among heteromorphic seeds and variations of heteromorphic ratio among different calendar years were presumed as diverse adaptation strategies integrated in the individual mother plant, and might apply important ecological significance for successful reproduction of the species in the unpredictable cold desert.

## Introduction

Seed germination is a crucial stage for angiosperm in population settlement and propagation, especially the annual herbs in heterogeneous circumstances (Volis, 2016). Seeds have to face a variety of abiotic stresses in natural habitats, e.g., salinity, drought, chilling, or heat, which is becoming the major constraints affecting seed germination (Jaganathan, 2016). The complex responses of seeds to stress involve in various morphological, physiological, and cellular changes (Tardieu and Tuberosa, 2010; Wang et al., 2015), which may contribute to their plasticity degree. The accurate prediction of the relationship between germination percentage and environmental responses of seeds in natural habitat has long become an objective of ecological specialist.

Heterocarpy or seed heteromorphism, in which seeds of different form or behavior are produced by single individual, is thought to be an adaptive strategy and plays a vital role in escaping from the negative effect of crowding and reducing sibling competition in natural habitats (Venable and Lawlor, 1980; Baskin and Baskin, 2014). It is a common phenomenon in a number of plant genera like *Arthrocnemum*, *Chenopodium*, *Cakile*, *Salicornia*, *Salsola*, *Spergularia*, *Suaeda*, *Trianthema*, *Atriplex*, in which many are halophytes (Gul et al., 2013). Heteromorphic seeds can differ in their external appearance, which fall into several categories, i.e., size, shape, color, or dispersal structure (Takeno and Yamaguchi, 1991; Imbert, 2002; Mandák and Pyšek, 2005). Winged pericarp (bracteole) is one of the obvious characteristics of seeds in species of *Salsola* (pericarp) or *Atriplex* (bracteole), and in which the seed heteromorphism is generated by the largely different size (radius) of the wing within a single plant (Wei et al., 2008). Seeds with winged perianth (WP) apparently take the advantage in dispersal (Baskin et al., 2014). However, much less is known about variations of seed heteromorphism in different positions within a dispersal unit (plant) (Koller, 1956; Wei et al., 2008; Volis et al., 2014). Seed heteromorphism may vary with the fluctuation of the natural habitat conditions in different calendar years, e.g., temperature, precipitation, soil nutrient, which can have great effects on seed morph ratio or size, germination or dormancy of heteromorphic seed (Wang et al., 2012; Gul et al., 2013; Lu et al., 2014). A lines of evidence on annual seed output was investigated for trends in seed abundance over time, which suggests that the seed production and heteromorphic seed ratio will ultimately be constrained by threshold high temperatures in the seed maturation year (Buechling et al., 2016).

Seed morphology of heteromorphic seeds is often associated with the variations in seed dormancy (Venable, 1985; Childs et al., 2010), which is another adaptation to heterogeneous environments of many wild seed plant populations (Mandák and Pyšek, 1999; Baskin and Baskin, 2014). Seed dormancy is usually caused by morphological (Hepher and Roberts, 1985; Vandelook et al., 2009), physical (Long et al., 2012), physiological (Cadman et al., 2006; Conversa and Elia, 2009) or combined factors (Baskin and Baskin, 1998; Wake and Fennell, 2000; Sun et al., 2009), which can be classified into different types. A cold stratification (CS) may suffice to overcome embryo dormancy caused by morphological constraint and non-deep physiological reasons in many plant species (Barton, 1965; Roberts, 1986). Phytohormones are known to play important roles in seed germination or dormancy (Li et al., 2008; Emamipoor and Maziah, 2014; Shu et al., 2016). Gibberellin (GA) with a concomitant effect on seed germination, which can break the deep dormancy aroused by physiological reasons, is investigated and a model presented to account for the characteristics of the dormancy mechanism (Conversa et al., 2010; Tuttle et al., 2015). Light is another environmental factor to apply effects on seed germination (Ballaré and Casal, 2000; Hameed et al., 2013; Zehra et al., 2013), which may cause the secondary conditional dormancy to light-sensitive plant seeds (Baskin et al., 1995). Germination of some halophyte seeds is completely inhibited in dark environment, while others are not sensitive to light (Gul et al., 2013). It has been reported that phytohormone as photoreceptor can mediate light regulation in initiating or stopping some physiological processes in seed dormancy (Brady and McCourt, 2003). In light-requiring species, the significant effect of light on germination may associate with phytochromes (Roberts, 1986; Pons and van der Toorn, 1988; Plummer and Bell, 1995). So far, however, the mechanism of light promoting or inhibiting seed germination is still less known (Motsa et al., 2015).

Seed germination is also affected by many other environmental factors, e.g., storage time, temperature fluctuations, salinity, drought, etc., especially for seeds with WP (bracteole), which have greater plastic responses (Gul et al., 2013). Seed germination and vigor can be significantly changed by after-ripening and temperature variation in protein storage, enzymatic activities and the biological metabolism (Rajjou et al., 2012; Bennett, 2015), which can consequently affect the germination behavior (Baskin and Baskin, 1998). Large temperature fluctuation exists between day and night in desert habitats, which forces desert plants to evolve with a wide temperature range in seed germination (Long et al., 2012), however, different species exhibit the diverse optimum regime (Cao et al., 2012; Gul et al., 2013; Gremer et al., 2016). *Salsola ferganica*, an annual desert pioneer halophyte, belongs to *Salsola* genus in Chenopodiaceae family distributed in extreme desert habitats in the north-western part of China (Wang et al., 2013; Ma et al., 2016), which has evolved special morphological structures against environmental stress (Sun et al., 2009; Yang et al., 2015), e.g., the whole plant is covered with white, long, soft hairs (trichomes) in seedling stage, the trichomes will become thinner at the later stage of plant development. In the present study, we found that *S. ferganica* could produce seeds with different sizes of WP which exhibit many different characteristics, especially in seed germination. However, little information has been documented on this halophyte species so far (Wang et al., 2013; He et al., 2016). To illustrate the unique characteristics of *S. ferganica* seed heteromorphism in germination and the ecological significance in adaptation to the desert environments, in the present study, we performed investigations in the following aspects: (i) to clarify seed heteromorphism of *S. ferganica* and the ecological significance of the WP on seed germination/dormancy and the dispersal ability; (ii) to analyze the dormancy type of *S. ferganica*, especially the sensitivity to light; (iii) to investigate the effects of environmental factors, e.g., day/night temperature variation, storage time, salinity on germination of heteromorphic seeds. Finally, we proposed a model for possible adaption strategy and the dynamics of seed reproduction for heteromorphic seeds of *S. ferganica* under cold desert conditions.

## Materials and Methods

### Source of Seeds

Mature seeds of *S. ferganica* were collected from a natural plant population growing in a heavy saline-alkaline wasteland at the edge of Junggar Basin, Xinjiang, China (44°19′N, 86°57′E; 429 mH) in September, 2013, October, 2014, and October, 2015. This area belongs to a typical continental semi-arid and arid climate with an annual precipitation of 100–250 mm, and the mean temperature of 27.5°C of the warmest month (July) and -26.7°C of the coldest month (January) based on the previous 8 years (2008–2015) data (provided by Xinjiang Meteorological Information Center). In the natural habitats, *S. ferganica* germinates in late March, blooms in middle of July, and bears fruits in September (observed data). Harvested seeds were air-dried under room conditions: 18–25°C, 10–25% relative humidity, then cleaned to remove the impurities, and stored in brown paper bag in basement for various experiments. Experiments were carried out during three successive years between October, 2013 and May, 2016.

### Morphology of Heteromorphic Seeds

A stereomicroscope SMZ800 (Nikon, Japan) was used to observe the heteromorphic seeds with or without WP and the morphology of germinated seed. Digital photographs were manipulated with Adobe Photoshop to prepare figures.

### Seed Heteromorphism Relevant Information

For calculation of heteromorphic seed percentage, mature seeds collected from different parts of the natural plant of *S. ferganica* were divided into large, middle or small seeds (SSs) depending on the size of the WP, the percentage of heteromorphic seeds was calculated by analyzing four plants (selected from 30 plants in original habitats with normal representative plant height and branch numbers) in 2013, 2014, and 2015, respectively. Poorly developed or shriveled seeds were excluded. For determination of 100 seed mass, seven replicates with 100 randomly chosen intact mature seeds of each from different parts of the plant and measured with a Sartorius BS210S electronic analytical balance (Sartorius Group, German). Radius of WP of 30 seeds for each type of heteromorphic seeds, or diameter of 30 seeds without WP was measured using digital calipers.

### Natural Habitat Conditions

#### Meteorological Data

**FIGURE 1:**
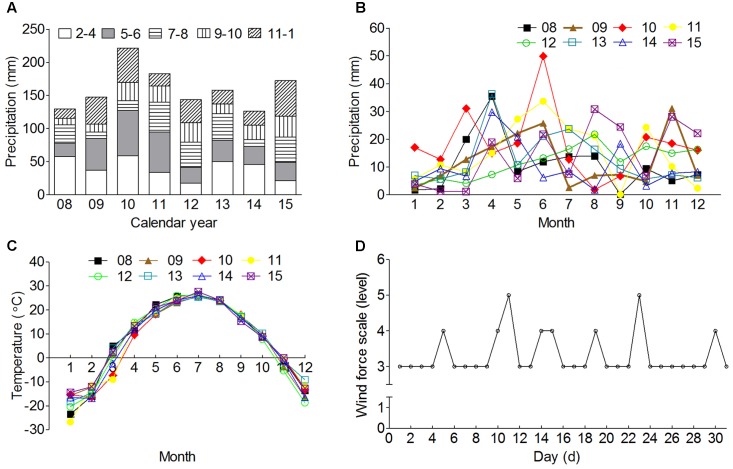
The information of precipitation, average temperature and wind force of the natural habitat of *S. ferganica.* **(A)** Annual precipitation from 2008 to 2015. 2–4, 5–6, 7–8, 9–10, or 11–1: represents precipitation from February to April in spring, May to June in early summer, July to August in summer, September to October in autumn, November to January in winter, respectively. **(B)** Monthly precipitation of whole year from 2008 to 2015. **(C)** Monthly average temperature of whole year from 2008 to 2015. **(D)** Daily wind force in October 2015. 08, 09, 10, 11, 12, 13, 14, and 15: represent calendar years of 2008, 2009, 2010, 2011, 2012, 2013, 2014, and 2015, respectively.

Data of seasonal precipitation, monthly precipitation, and monthly average temperature of previous eight calendar years (2008–2015) of the natural habitats of *S. ferganica* were obtained from Xinjiang Meteorological Information Center (Urumqi, Xinjiang, China). The data of daily wind force scale (level) in October 2015 were retrieved from the website of Weather China^1^. A general analysis was performed with these data (**Figure 1**).

#### Soil Components

Four replicates of the soil samples were collected from the natural habitats of *S. ferganica* at Wujiaqu 103 regiment (44°29′821″ N, 87°31′181″ E), Xinjiang, China. Samples from different depths (0, 5, 10, and 15 cm) of the soil were taken. The components and chemical and physical properties of the soil sample were analyzed by Xinjiang Institute of Ecology and Geography, Chinese Academy of Sciences (Urumqi, Xinjiang, China), such as soil organic carbon, electric conductivity, pH value and total salts. In addition, the content of CO_3_^2-^, HCO_3_^-^, Cl^-^, SO_4_^2-^ or Ca^2+^, Mg^2+^, Na^+^, K^+^ were determined by ion chromatograph (ICS-5000, Thermo Fisher Scientific, United States) and the inductively coupled plasma emission spectrum (735 ICP-OES, Agilent, United States), respectively (**Table 1**).

**Table 1:** The physical and chemical properties of the natural habitat soil of *S. ferganica.*

Depth (cm)	Organic carbon (mg/g)	Conductance (ms/cm)	pH	Ion content (mg/g)					
				
				Total salt	CO_3_^2-^	HCO_3_^-^	Cl^-^	SO_4_^2-^	Ca^2+^	Mg^2+^	Na^+^	K^+^
0	13.10	32.28	8.74	138.90	0.02	0.46	0.01	88.18	2.78	0.84	37.66	0.03
5	14.82	44.15	8.87	181.80	0.04	0.55	0.01	116.40	3.88	0.76	50.00	0.18
10	17.60	8.88	8.86	41.87	0.03	0.41	2.51	23.36	1.47	0.28	10.73	0.12
15	21.56	8.26	8.83	36.58	0.03	0.37	3.02	19.17	1.00	0.33	9.48	0.10


### Seed Dispersal Experiments

#### Seed Dispersal in Natural Habitat

Six plants in distance of 15 m from each other were selected in the natural habitat of *S. ferganica*, and the WP of all seeds on each plant was stained with 0.1% Safranin or Fast Green dye just before seed maturation. One week later, the seed dispersal number of four circles with radius of 0.2, 0.5, 1.0, and 3.0 m around each mother plant was investigated.

#### Indoor Seed Dispersal Experiment

Dispersal radius was determined following the method of Lu et al. (2010) and Mamut et al. (2014). Three replicates of randomly selected seeds with one thousand of each were exposed to a continuous stream of air (generated by one or two fans) paralleled to the flat diaspore-landing surface for 60 s. Seeds were released at a height of 30 cm and exposed to wind velocity of 1.8–2.2 m⋅s^-1^ (light wind), 2.8–3.8 m⋅s^-1^ (gentle wind), and 4.1–4.8 m⋅s^-1^ (mild wind) based on the data presented in **Figure 1**. Seeds dispersed of four circles with radius of 0.2, 0.5, 1.0, and 3.0 m around were investigated.

### Seed Germination Experiments

Four replicates with 25 seeds of each of heteromorphic seeds were tested in germination experiment. Seeds were placed on a double layers of wet filter paper within a 9 cm Petri dish, which was added 7 mL distilled water. All Petri dishes were sealed with cling film and placed in an illuminated incubator (RXZ-500D-LED; Jiangnan Apparatus Manufactory, China), and subjected to a for a 12-h daily light period under constant illumination at 100 μmol m^-2^ s^-1^ at 25°C. The relative humidity was approximate 30–40%. For seed germination in dark, Petri dish was wrapped and sealed with foil paper under green color light in dark room and would not be uncovered until the end of the experiment. A seed was considered to be germinated when the radicle was at least 1 mm. Non-germinated seeds were stained by TTC (2,3,5-triphenyltetrazolium chloride) and checked under a stereomicroscope to see if the embryos were stained (alive); only viable seeds were considered in further calculation (Baskin and Baskin, 1998).

#### Winged Perianth Removal Treatments

To investigate the effect of WP on germination of heteromorphic seeds, seeds were carefully removed with WP but remained the inner membrane intact. Heteromorphic seeds with or without WP were employed in various tests.

#### Cold Stratification

Four replicates of 50 intact heteromorphic seeds (with WP; collected in 2014) of each were stratified on moist filter paper at 4°C for 1, 3, 5, 7, 14, or 21 days, then transferred to germination test.

#### Different Wavelength Light (DWL) Treatments

To determine the effect of DWL on germination of seeds [only tested with large seeds (LSs)], blue (450–490 nm wavelength), green (491–570 nm), far-red (570–621 nm), yellow (570–750 nm) and red (621–750 nm), and lights were employed in our test, and white (380–750 nm) light and darkness were used as two controls. DWLs were simulated by the special transparent color paper (thickness: 0.036 mm; Libao Decoration Material Limited Liability Company, Changshu, China). Before Petri dishes were sealed in cases made of monolayer of above transparent color paper, seed sowing was performed under lamps covered with the corresponding color paper in a dark room. To further investigate the time effect of DWL, varying time periods (1, 3, 5, or 14 days) of blue, green, far-red, yellow, red, white light or darkness were applied to seed germination, and then the recovery treatment was conducted by transferring the Petri dishes to white light for another 7 days.

#### GA_3_ Treatments

To determine the response (sensitivity) to GA_3_ of heteromorphic seeds with or without WP, seeds of each type were sown on the plate containing 0, 1, 5, 10, 50, or 500 mM of GA_3_ for germination assays.

#### Night/Day Temperature Variation (N/DTV) Treatments

To investigate the effect of daily temperature variation on germination of heteromorphic seeds, N/DTV was applied for the tests. Four ranges of temperature variation between night and day were designed in our test as: 5°C (in night)/15°C (in day), 10°C/20°C, 15°C/25°C, and 20°C/30°C, which were employed to simulate temperature changes in early spring, spring, later spring and summer, respectively, in the natural habitat. 25°C (in night)/25°C (in day) was used as control.

#### Salinity Treatments

Two types of salt were employed in our test according to analysis of soil in natural habitat of *S. ferganica* (**Table 1**), different concentrations of NaCl or Na_2_SO_4_ (0, 100, 300, 500, 700, and 1000 mM) were applied in seed germination experiment. After 2-week germination, the recovery experiment was performed by rinsing non-germinated seeds three times with distilled water and then set to germinate in distilled water for another 7 days.

### Ion Concentration and Phytohormone Level of Winged Perianth (WP)

The leaching aqueous solution of WP was prepared for determination of the concentration of ions and phytohormones. For ion measurement, WP of 50 seeds were submerged in 10 mL deionized distilled water and the mixture was continuously stirred for 10 min at 30°C, then left at 30°C for 3–4 h. Before treated with 0.45 μm filter, the leaching solution and WP mixture was stirred for another 3–5 min. Four ions of K^+^, Na^+^, Ca^2+^, Mg^2+^ of the leaching solution were determined by Flame Atomic Absorption Spectrometry (Agilent AA240 atomic absorption spectrometer, United States). Standard curve of four ions was generated from the standard solution and the series dilutions, with which the ion concentration of sample was calculated. Three biological replicates were applied in the test. For measurement of two phytohormones – ABA (Abscisic acid) and GA_3_ of WP, three replicates of 50 WP of each were homogenized in liquid nitrogen and transferred into 10 mL deionized distilled water at 30°C for 3–4 h to extract the phytohormones. The leaching solution was pre-treated according to the method described by Hou et al. (2008). High performance liquid chromatography (HPLC1100, Agilent Technologies, United States) was employed in phytohormone analysis. Standard curves were generated from the standard solutions of GA_3_, ABA, and their series dilutions. Measurement of the concentration of ABA or GA_3_ was based on the method described by Zhang et al. (2005). Conditions for chromatography were: (A) Mobile phase: methanol: glacial acetic acid (1% in H_2_O) = 4:6; (B) Flow rate: 1.0 mL/min; (C) Column temperature: 35°C; (D) Detection wavelength: 252.4 nm.

### Statistical Analysis

All data were expressed as mean ± standard error. Germination percentage was arcsine transformed to ensure homogeneity of variance. One- or two-way ANOVA was used to analyze data collected from the effect of WP removal, night/day temperature variation, GA_3_, salinity treatments on seed germination experiments using the GraphPad Prism version 6.01 for Windows (GraphPad Software, San Diego, CA, United States). Three-way ANOVA was employed to compare the effect of different wavelength light treatment on seed germination using the SPSS version 17.0 for Windows (SPSS Inc., Chicago, IL, United States). When significant main effects existed, differences were tested by a multiple comparison Tukey test at 0.05, 0.01, 0.001, and 0.0001 significance levels.

## Results

### Characterization of Seed Heteromorphism of *S. ferganica*

#### Morphology

**FIGURE 2:**
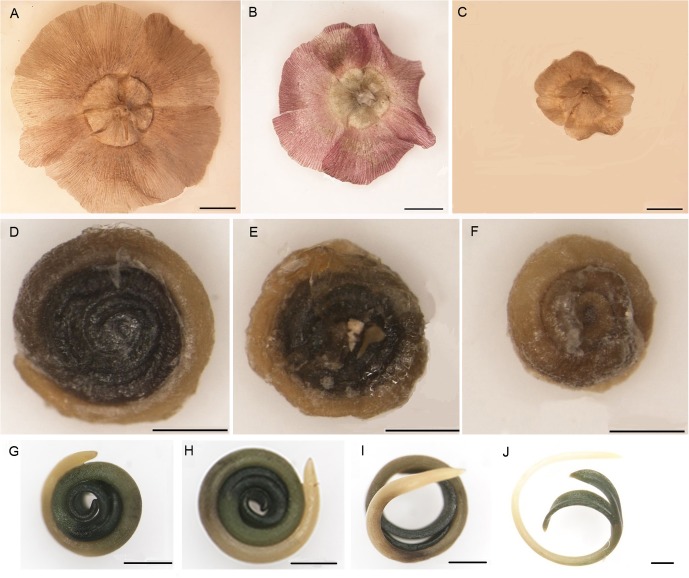
Morphology of heteromorphic seeds of *S. ferganica*. **(A–F)** Intact dry seeds. **(A)** Large seed with winged perianth (WP). **(B)** Middle seed with WP. **(C)** Small seed with WP. **(D)** Large seed without WP. **(E)** Middle seed without WP. **(F)** Small seed without WP. **(G–J)** Germination process of spiral embryo of seed without WP. Scale bars: **(A–J)**: 1 mm.

*Salsola ferganica* produces nut as fruit, but it was summarized in the term “seed” with referring to other reported work (Takeno and Yamaguchi, 1991; Wei et al., 2008; Wang et al., 2013), which differ in WP morphology (**Figure 2**) and germination behavior. According to the significant differences of WP radius, seed diameter, 100-seed mass (**Table 2**), *S. ferganica* seeds were divided into large (**Figures 2A,D**), middle (**Figures 2B,E**) and small (**Figures 2C,F**) types. They had the typical spiral embryo (**Figures 2G–J**) as many desert plant species.

**Table 2:** The characteristics of heteromorphic seeds of *S. ferganica.*

Seed size	Radius of WP (mm)	Diameter of NWP seed (mm)	Mass/100 seeds of WWP (g)	Mass/100 seeds of NWP (g)
Large	4.54 ± 0.04^a^	2.58 ± 0.02^a^	0.85 ± 0.01^a^	0.48 ± 0.01^a^
Middle	3.23 ± 0.03^b^	2.33 ± 0.04^b^	0.72 ± 0.07^b^	0.39 ± 0.01^b^
Small	1.82 ± 0.09^c^	1.91 ± 0.03^c^	0.50 ± 0.01^c^	0.25 ± 0.01^c^


*WP, winged perianth; NWP, without winged perianth; WWP, with winged perianth.*

**FIGURE 3:**
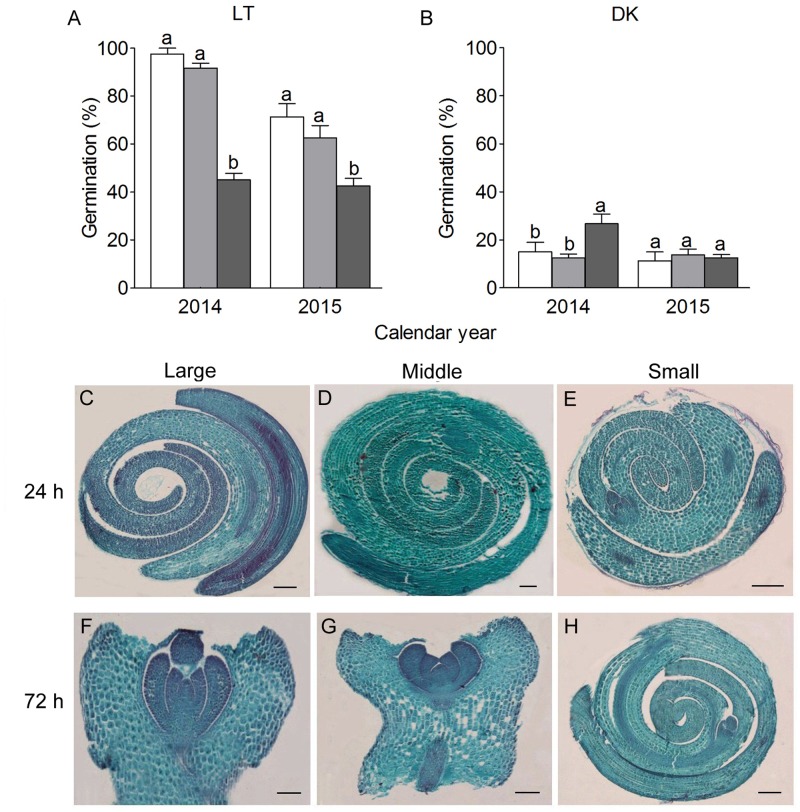
Seed germination and the morphological changes of heteromorphic seed of *S. ferganica.* **(A,B)** Germination percentage of heteromorphic seeds in light or dark in February of the next year of 2014 and 2015, respectively. Bars with different lowercase letters indicate significant differences (*P* < 0.05) according to the Tukey’s test. Values are means ± SE of four replicates in **(A,B)**. **(C–H)** Longitudinal cross section of seed; **(C,F)** large seed; **(D,G)** middle seed; **(E,H)** small seed. **(C–E)** Germination for 24 h. **(F–H)** germination for 72 h. Scale bars: **(C–E)** 200 μm, **(F–H)** 100 μm.

The germination behavior of three types of seed differed significantly (**Figures 3A,B**): the LSs had highest germination percentage (GP) while the SSs had the lowest GP, the middle ones were in between but much closer to the LSs. GP of heteromorphic seeds presented significant variations between 2014 and 2015 calendar years, especially the large and middle seeds (MSs). Furthermore, we observed that germination of *S. ferganica* seeds displayed apparently light-sensitive effect, which has been further analyzed in the following experiments.

To investigate the morphological change of different types of seed in germination, paraffin sections were prepared and inspected under microscope. Results showed that significant difference with the embryo change existed among three types of seed in early germination process (**Figures 3C–H**). After 24 h germination, the spiral embryo of LS began to stretch, and the true leaf primordia developed quicker than those of the middle or SS (**Figures 3C–E**). Seventy-two hours later, both large and MSs fully germinated, several true leaves were apparently seen at the apical growth point of the large or MSs (**Figures 3F,G**), while the eldest true leaf of LS was much longer than that of the MS (0.43 ± 0.002 mm vs. 0.32 ± 0.023 mm). However, the SS still kept ungerminated state (**Figure 3H**) at this moment.

#### Variations of Seed Heteromorph Ratio among Different Calendar Years

**FIGURE 4:**
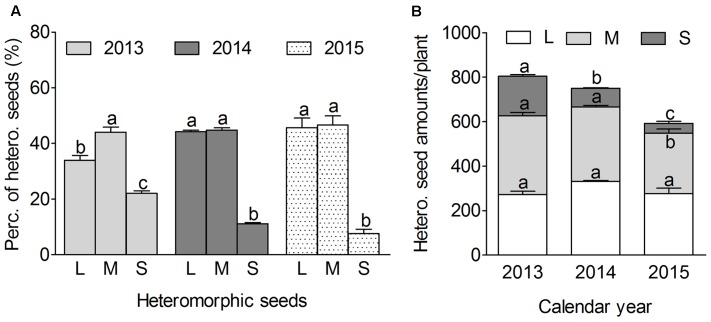
Variation of seed heteromorphic ratio of *S. ferganica* collected from 2013, 2014 and 2015 calendar years in natural habitats. Different plants of *S. ferganica* randomly chosen at early stage of seed maturation for calculation of the ratio of heteromorphic seeds. Perc., percentage; Hetero., heteromorphic; No., number; L, M, S, large, middle, small seed; LT, light; DK, dark. **(A)** Percentage of heteromorphic seeds. **(B)** Heteromorphic seed number per plant. Bars with different lowercase letters indicate significant differences (*P* < 0.05) according to the Tukey’s test. Values are means ± SE of four replicates.

Analysis of the percentage of heteromorphic seed showed that the middle or LSs accounted for higher proportion than that of the SS in the same or different calendar years (**Figure 4A**), variation was observed with seed heteromorphic ratio among different calendar years, especially for the large or SS. The LS percentage was similar or lower than the middle one, and which seemed to present opposite correlation with the SS, i.e., the LS had the lower proportion in 2013 while higher in 2015; whereas the SS showed the higher proportion in 2013 while lower in 2015. Besides, the highest seed number per plant was in 2013, while the least in 2015 (*P* < 0.001) (**Figure 4B**), it suggests that SS proportion tends to increase while LS proportion tends to decrease with the rising of total seed number per plant.

### Seed (Fruit) – Setting and Dispersal Patterns of Heteromorphic Seeds of *S. ferganica*

#### Seed (Fruit)-Setting Pattern

**FIGURE 5:**
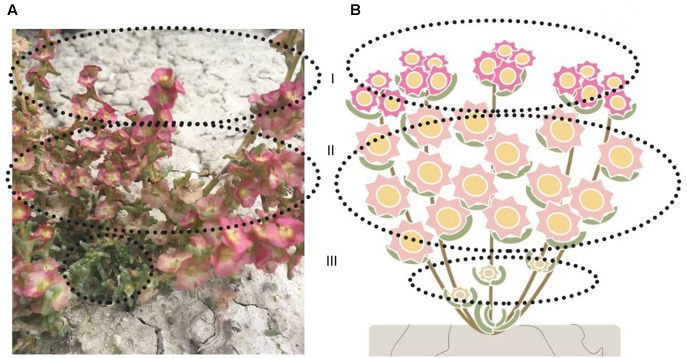
Schematic diagram of seed (fruit) setting pattern of heteromorphic seeds (fruits) of *S. ferganica.* **(A)** Picture of plant in fruit-bearing stage in its natural habitat. **(B)** Schematic diagram of fruit-bearing pattern. **(I)** The top part of the plant (branches), mainly including medium-sized seeds. **(II)** The middle part of the plant (branches), mainly including large-sized seeds. **(III)** The base part of the plant (branches), mainly including small-sized seeds. The dashed-line circle indicates different part of the plant.

*Salsola ferganica* is an annual herb with the plant height of 10–30 cm, large number of branches are generated from the base of the stem, the compact architecture makes the plant look like a ‘round ball.’ Fruits are born from August to September, which showed different patterns at different parts of the branches (**Figure 5**), i.e., at the top of the branch (which is the outmost layer of the plant), medium-sized seeds were distributed [**Figures 5A(I),B(I)**]; the middle part (which is the inner part of the plant) distributed large-sized seeds [**Figures 5A(II),B(II)**]; the base part was small-sized seeds located [**Figures 5A(III),B(III)**].

#### Seed Dispersal Pattern

**FIGURE 6:**
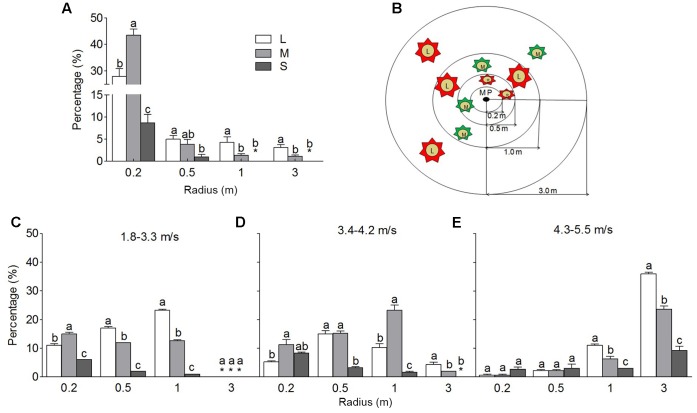
Dispersal patterns of heteromorphic seeds of *S. ferganica* surrounding mother plant in natural habitat or under control wind force in laboratory. Four circles with radius of 0.2, 0.5, 1.0, and 3.0 m were established around each mother plant, the number of seeds dispersed into each circle were recorded and the proportion of heteromorphic seeds in mother plant was calculated. **(A)** Percentage of heteromorphic seeds dispersed into different zones surrounding mother plant in natural habitat. **(B)** Schematic diagram of dispersal pattern of heteromorphic seeds. L, M, S: large, middle, small seed. 0.2, 0.5, 1.0, and 3.0 m: circles with radius of 0.2, 0.5, 1.0, and 3.0 m. **(C–E)** Artificial seed dispersal experiment, showing ten thousand of heteromorphic seeds were dispersed into four different radius circles at 0.2, 0.5, 1.0, and 3.0 m in laboratory, and the proportion of heteromorphic seeds was calculated. 1.8–3.3, 3.4–4.2, and 4.3–5.5 m⋅s^-1^: different level of wind velocity. ^∗^ represents the value of zero. Bars with different lowercase letters indicate significant differences (*P* < 0.05) according to the Tukey’s test. Values are means ± SE of six replicates in **(B)**, or four replicates in **(C–E)**.

To investigate the dispersal ability, we tested the heteromorphic seeds of *S. ferganica* for 1 week in the natural habitat in October, 2015 (**Figure 6A**), results showed that the largest proportion of seeds (more than 85%) was observed within 0.5 m radius to mother plant, and with the distance increasing, seed amounts significantly decreased, while the LS proportion was generally significantly higher than the middle one, the SS could hardly be detected. To verify such a trend, we performed the dispersal experiments of the heteromorphic seeds in laboratory (**Figures 6C–E**). In the light or gentle wind (1.8–3.3 m/s or 3.4–4.2 m/s), most of the seeds were dispersed within 1 m circle, and fewer seeds were seen in 3 m circle (**Figures 6C,D**). While in the mild wind (4.3–5.5 m/s), most of seeds were dispersed within 3 m circle, and the least of seeds appeared in 0.2 m circle (**Figure 6E**). Our results showed that, no matter the wind velocity was lower or higher, the LS always occupied the largest proportion in total at the farthest location. Based on the observation in the field and laboratory, a schematic diagram was proposed as **Figure 6B**, in which the heteromorphic seeds were sorted in different radius circles with different proportion, the dispersal distance was positively related to the size of the WP.

### Classification of Seed Dormancy of *S. ferganica*

#### Light Sensitivity

**FIGURE 7:**
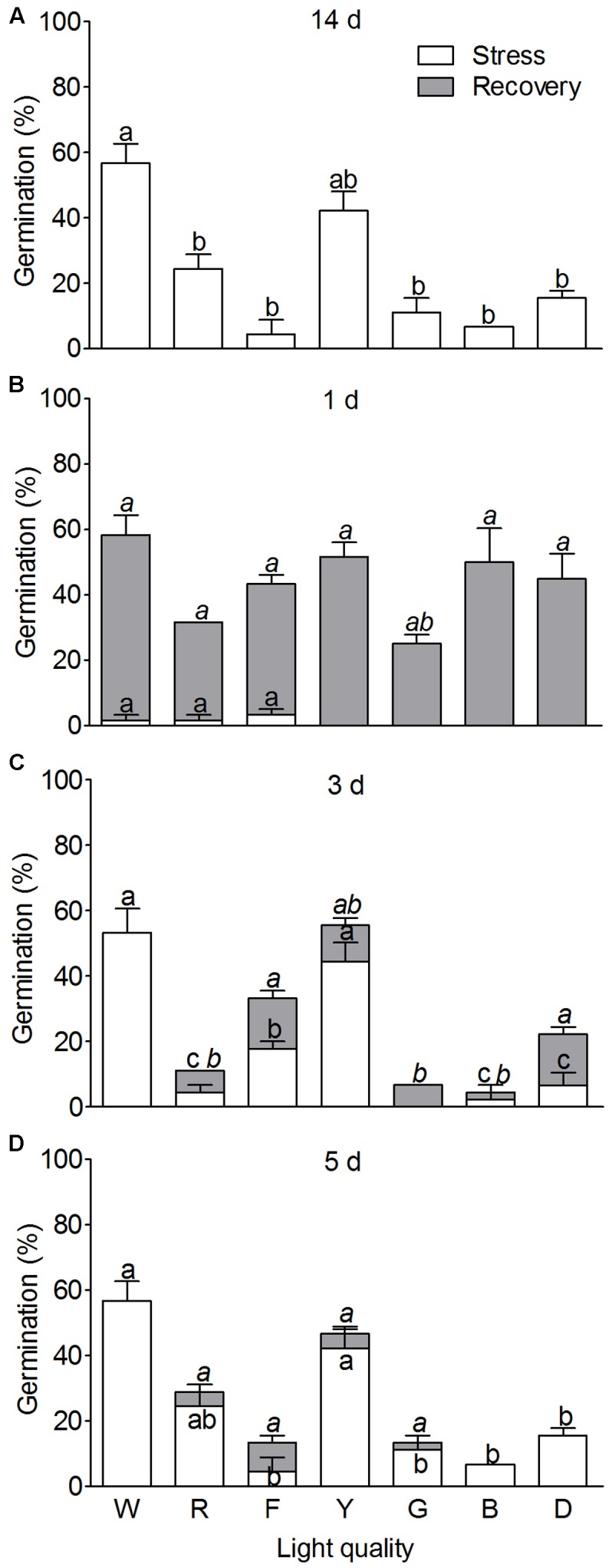
The effect of different wavelength light on seed germination of *S. ferganica*. Only large seeds were tested in this experiment. NWP, non-winged perianth; WWP, with winged perianth. W, R, F, Y, G, B, D: white, red, far red, yellow, green, black, dark light. **(A–D)** Different wavelength light treatment for 1, 3, and 5 days, respectively, and their recovery. 1, 3, and 5 d: Different wavelength light treatment for 1, 3, and 5 days, respectively. Stress: Treated with different wavelength light. Recovery: Transferring seeds into white light treatment. Bars with different lowercase letters indicate significant differences (*P* < 0.05) according to the Tukey’s test. Values are means ± SE of four replicates.

Compared to the control (white light), the monochromatic light applied distinct effects on seed germination (SG) of *S. ferganica* (**Figure 7A**). Except for the yellow light, red, far-red, green, and blue lights including darkness all had significantly negative effects on SG. Our experiments indicate that SG of *S. ferganica* was light-sensitive (*P* < 0.0001) (**Table 3**). To clarify the time effect of different wavelength light (DWL) on SG, we treated the LSs with DWLs. Results showed that the recovery ability of seed germination significantly decreased with treatment time increasing from 1 to 5 days (**Figures 7B–D**), moreover, short time treatment with DWL could significantly promote seed germinability when recovered under normal conditions, analysis of DWL, treatment time and their interaction on SG showed significant difference (*P* < 0.0001) (**Table 3**). Our results indicate that light-sensitive of *S. ferganica* seed happened in the first few days of germination, and the germination percentage could not be recovered any more after that time.

**Table 3:** Two-way ANOVA of effects of different wavelength light, treatment time and their interaction on seed germination of *S. ferganica.*

Source of variation	Df.	*F*-value	*P*-value
Different wavelength light (L)	6	36.128	<0.0001
Time (D)	3	64.600	<0.0001
L × D	18	6.953	<0.0001


#### Winged Perianth

**FIGURE 8:**
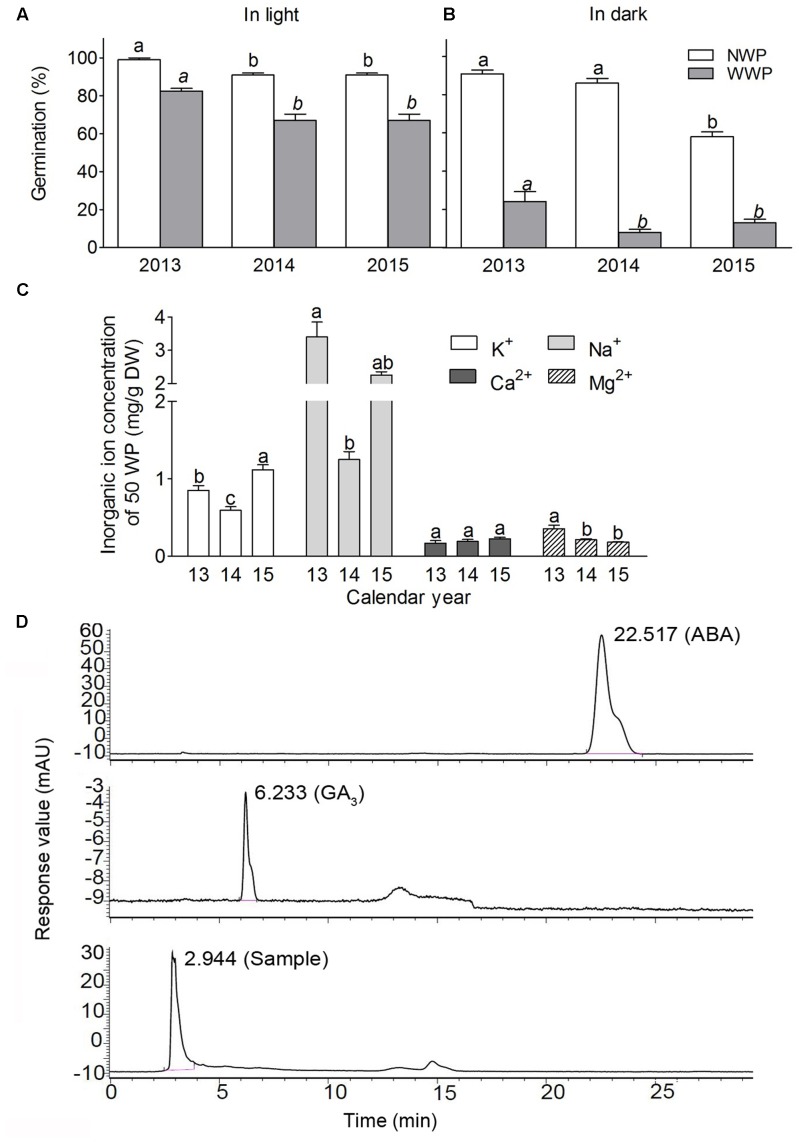
Effect of winged perianth (WP) on germination and analysis of ions and phytohormone of WP of heteromorphic seeds of *S. ferganica*. **(A)** Germination in light. **(B)** Germination in dark. 2013, 2014, 2015: Calendar year of seed collection. NWP, non-winged perianth; WWP, with winged perianth. **(C)** Ion concentrations of winged perianth. Ions were extracted from 500 pieces of winged perianth with 100 mL deionized distilled water. CK: deionized H_2_O; 13, 14, 15: 2013, 2014, 2015 calendar year. **(D)** Chromatographic curve of two phytohormones. Upper panel: ABA standard product, ABA peak appeared at 22.517 min; middle panel: GA_3_ standard product, peak at 6.233 min; lower panel: sample extracted from WP, peak at 2.944 min. Bars with different lowercase letters indicate significant differences (*P* < 0.05) according to the Tukey’s test. Values are means ± SE of four replicates.

Our experiment showed that WP applied significant effect on seed germination. When WP being removed (NWP), heteromorphic seeds presented significantly higher germination percentage than seeds with winged perianth (WWP) (**Figures 8A,B**), especially under darkness (**Figure 8B**) (*P* < 0.001), moreover, the effect of WP on germination was variable among different calendar years. To gain insight into the reason why WP inhibited germination, we did the further analysis of WP on the content of total ions and phytohormones. Results showed that the content of total ions of WP was about 5 mg⋅g^-1^ from 50 WP (**Figure 8C**). The content of sodium ion was the highest as about 3 mg⋅g^-1^, other ions were about or lower than 1 mg⋅g^-1^, and ions were variable among different calendar years. In addition, results showed that ABA and GA_3_ from 50 WP (in 10 mL) were undetectable at the level of 10^-3^ mg⋅L^-1^ of standard hormone for the content of phytohormones analysis (**Figure 8D**).

#### Cold Stratification

**FIGURE 9:**
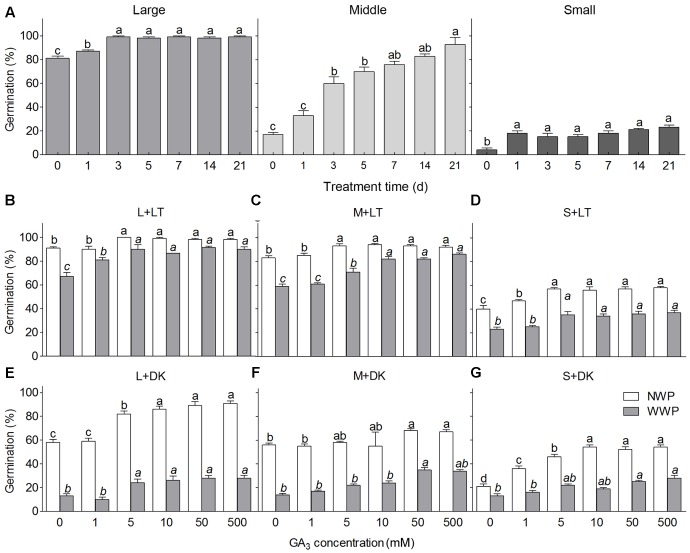
Effect of cold stratification **(A)**, GA_3_ treatment **(B–G)** on germination of heteromorphic seeds of *S. ferganica.* L, M, S, large, middle, small seed. LT, light; DK, dark. NWP, non-winged perianth; WWP, with winged perianth. Bars with different lowercase letters indicate significant differences (*P* < 0.05) according to the Tukey’s test. Values are means ± SE of four replicates.

Our experiment showed that CS could significantly promote germination of heteromorphic seeds (*P* < 0.05, *P* < 0.001, *P* < 0.01 for large, middle, SS, respectively), especially the MS, which showed a time-dependent effect (**Figure 9A**). One and/or three days of CS had the greatest enhancement on germination of heteromorphic seeds, while the effect was variable to different types of seed: with the time increasing, germination of LS increased from about 80% to nearly 100%; that of MS increased from less than 20% to more than 90%, while that of SS only increased from about 5% to around 20%.

#### GA_3_

Results showed that low GA_3_ concentration (1–10 mM) had significant effect on germination of heteromorphic seeds of *S. ferganica* (*P* < 0.001), no matter the seeds with or without winged perianth (WWP or NWP) (**Figures 9B–G**). Under light, GA_3_ applied greater effect on germination of WWP, while weaker promotion on seeds of NWP (**Figures 9B–D**); under darkness, however, GA_3_ showed the opposite effects between WWP and NWP (**Figures 9E–G**).

### Effects of Different Environmental Factors on Seed Germination of *S. ferganica*

#### Night/Day Temperature Variation (N/DTV)

**FIGURE 10:**
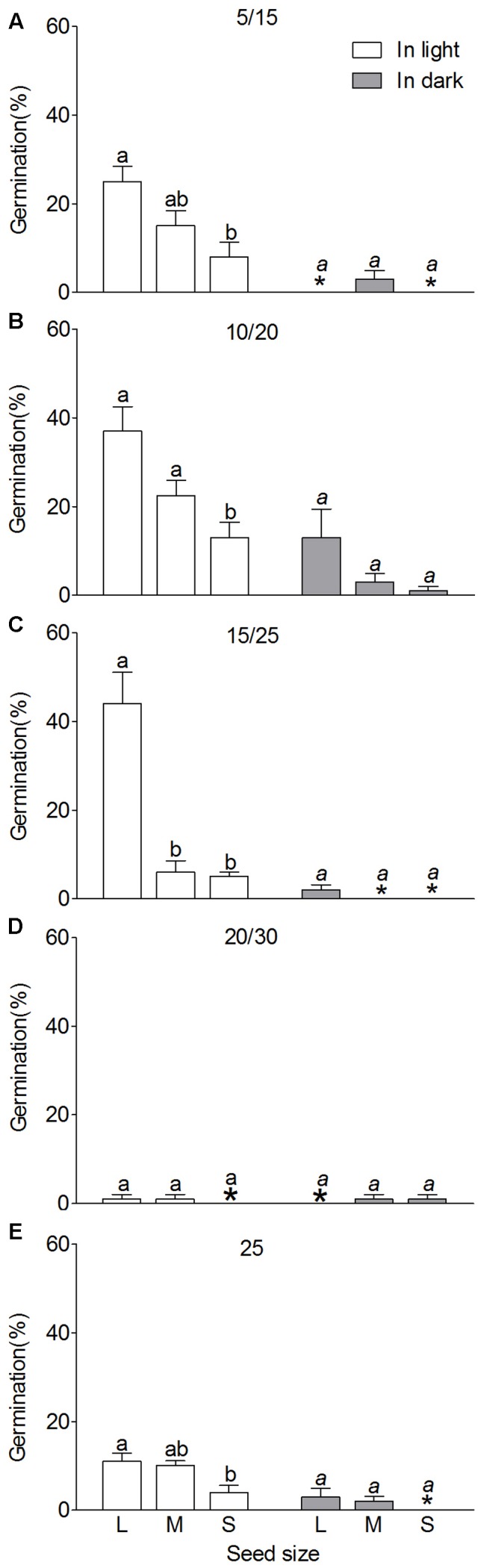
Effect of night/day temperature variation (N/DTV) on germination of heteromorphic seeds of *S. ferganica.* Seeds collected in 2014 were used in the test and seed vigor was lower at the moment. **(A–E)** Heteromorphic seeds incubated at different N/DTV. 5/15, 10/20, 15/25, 20/30, and 25: 5/15 represents 5°C in dark for 12 h and then 15°C in light for 12 h, and the same meaning for 10/20, 15/25, 20/30; 25 represents 25°C in dark for 12 h then in light for 12 h. ^∗^ represents the value of zero. L, M, S: large, middle, small seed. Bars with different lowercase letters indicate significant differences (*P* < 0.05) according to the Tukey’s test. Values are means ± SE of four replicates.

Night/day temperature variation significantly influenced germination of heteromorphic seeds of *S. ferganica* (**Figure 10**). Compared to the control (25°C/25°C), the relatively lower daily temperature range, i.e., 5°C/15°C, 10°C/20°C, or 15°C/25°C could enhance germination of heteromorphic seeds either in light or in dark (**Figures 10A–C**), especially at medium low temperature (10°C/20°C); whereas relatively higher N/DTV, i.e., 20°C/30°C significantly inhibited seed germination (**Figure 10D**). Analysis of effects of N/DTV (V), seed type (S), different wavelength of light (L) and their interactions on seed germination showed that, except for interaction of V × S × L, all effects were extremely significant difference (*P* = 0.003 or *P* < 0.0001) (**Table 4**).

**Table 4:** Three-way ANOVA of effects of night/day temperature variation (N/DTV), seed type, light and their interactions on seed germination of *S. ferganica.*

Source of variation	df.	*F*-value	*P*-value
N/DTV (V)	4	24.412	<0.0001
Seed type (S)	2	26.162	<0.0001
Light (L)	1	155.449	<0.0001
V × S	8	4.565	<0.0001
S × L	2	6.230	0.003
V × L	4	11.677	<0.0001
V × S × L	8	2.012	0.054


#### Duration of Storage at Room Temperature

**FIGURE 11:**
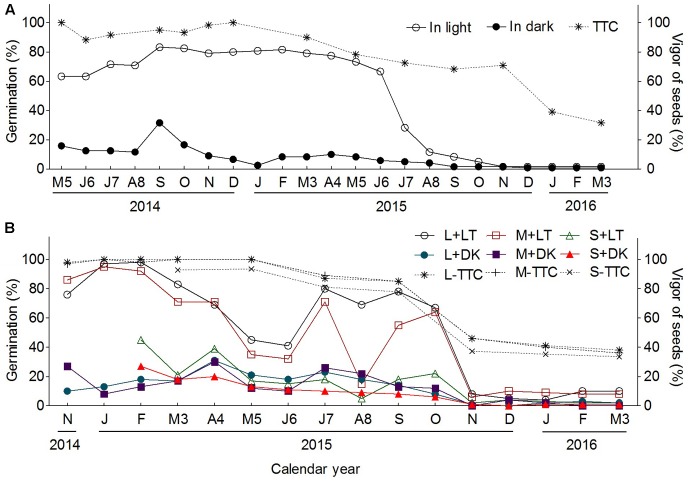
Effect of room storage on seed germination and analysis of seed vigor of *S. ferganica.* **(A)** Seeds collected from 2013; **(B)** seeds collected from 2014. 2014, 2015, 2016: represent seed germination performed in 2014, 2015, 2016 calendar year, respectively. J, January; F, February; M3, March; A4, April; M5, May; J6, June; J7, July; A8, August; S, September; O, October; N, November; D, December. L, M, S, large, middle, small seed. LT, light; DK, dark. TTC: 2,3,5-triphenyltetrazolium chloride.

Seeds collected in 2013 and 2014 calendar years differed in germination behavior spanning 3 years of storage time at room temperature (**Figure 11**). Seeds from 2013 could maintain higher germination percentage (60–80%) (under light rather than darkness) at least for 15 months from May of 2014 to July of 2015 (not including the 6 months from November of 2013 to April of 2014 which were not tested in the present study) (**Figure 11A**), furthermore, the TTC test suggested another 6 months higher vigor lasted from August of 2015 to January of 2016; however, the large and MSs from 2014 just kept higher germination (70–100%) for about 12 months from November of 2014 to October of 2015 under light (**Figure 11B**), with fluctuation in May, June, and August of 2015, which may be forced into secondary dormancy in summer hot weather; results of TTC test also suggested a significant decline of seed vigor from September to November of 2015; the SSs under light and three types of heteromorphic seed under darkness all maintained at a quite low germination percentage (20–40%) from November of 2014 to October of 2015. In comparison of seeds collected from these 2 years, we noticed that germination percentage of seeds from 2014 (80–100%) was much higher than that of 2013 (60–80%), whereas the germination percentage of seeds from both years all decreased suddenly from much higher to nearly zero, without any gradual change in between.

#### Salt Stress

**FIGURE 12:**
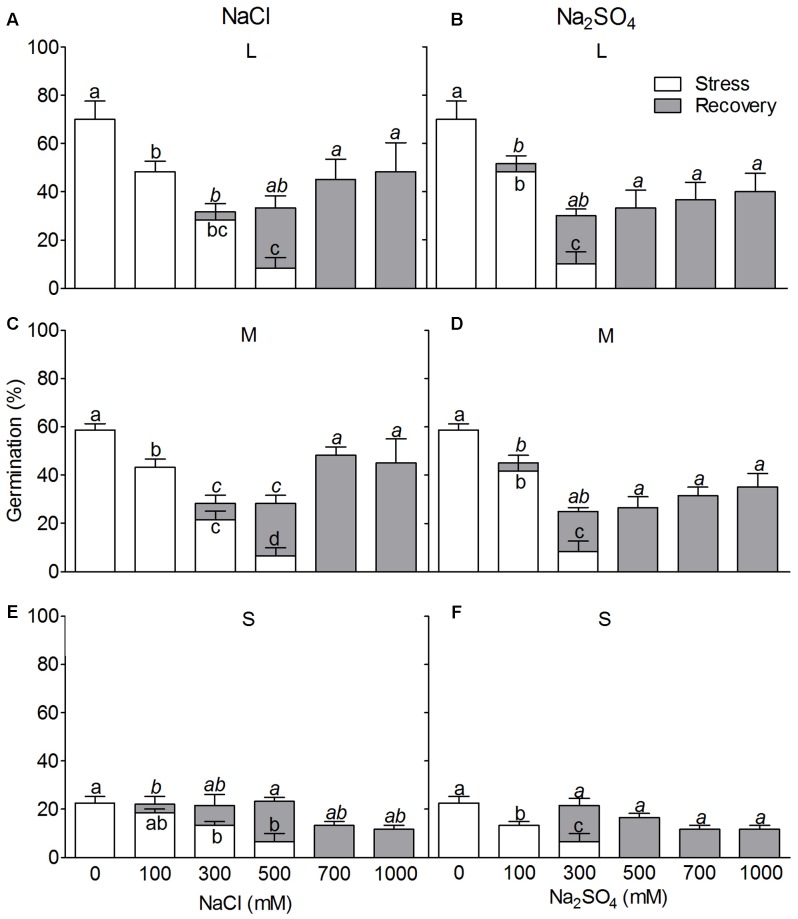
Effect of different salt types on germination of *S. ferganica* heteromorphic seeds. **(A–C)** NaCl treatment; **(D–F)** Na_2_SO_4_ treatment. **(A,D)** Large seed; **(B,E)** middle seed; **(C,F)** small seed. L, M, S, large, middle, small seed. Stress: salt treatment. Recovery: ungerminated seeds under salt treatment transferred into distilled water. Bars with different lowercase letters indicate significant differences (*P* < 0.05) according to the Tukey’s test. Values are means ± SE of four replicates.

We tested two types of salt – NaCl and Na_2_SO_4_ in seed germination according to the major components of the soil in the natural habitat of *S. ferganica.* Results showed that both NaCl and Na_2_SO_4_ could significantly decrease germination percentage of heteromorphic seeds (**Figure 12**), and the latter applied more negative effect (**Figures 12D–F**). Upon transferring into distilled water, ungerminated seeds under both salt treatments could be recovered to a moderate germination percentage, especially at relatively higher salt concentration (700 or 1000 mM), which suggest that inhibition of germination of heteromorphic seeds by ion can be reversible upon improved conditions. Analysis of effect of salt type (St), salt concentration (Sc), seed type (S) and their interactions on heteromorphic seed germination showed that St, Sc, S, and St × Sc, Sc × S all reached to extremely significant difference (*P* < 0.0001) (**Table 5**).

**Table 5:** Three-way ANOVA of effects of salt type, salt concentration, seed type and their interactions on seed germination of *S. ferganica.*

Source of variation	df.	*F*-value	*P*-value
Salt type (St)	1	19.425	<0.0001
Salt concentration (Sc)	5	223.838	<0.0001
Seed type (S)	2	29.439	<0.0001
St × Sc	5	6.455	<0.0001
St × S	2	0.056	0.945
Sc × S	10	8.536	<0.0001
St × Sc × S	10	0.203	0.995


## Discussion

As an annual halophyte in the extremely harsh environments, *S. ferganica* can produce heteromorphic seeds with different dispersal ability and germination behavior. So far, little has been reported on the heteromorphism and the characteristics of seed germination on this species. In the present study, we provided a systematical investigations of *S. ferganica* on seed heteromorphism definition, seed-setting and dispersal patterns, dormancy type, and germination characteristics of heteromorphic seeds. Results indicated that three types of seed, defined by the size of WP, showed significantly diverse seed-setting pattern and dispersal properties, and it belonged to photo-sensitive combined with morphological (WP) and non-deep physiological type of dormancy, in which light was the dominant factor. Not like other desert plant species, the germinability of the heteromorphic seeds of *S. ferganica* could not sustain for long (only 1–2 years), especially the SSs, and was affected by storage time, temperature variation, salinity. Thus, the differences existed among heteromorphic seeds, the light sensitivity, and variations of heteromorphic ratio among different calendar years were presumed as diverse adaptation strategies integrated in the individual mother plant, and might apply important ecological significance for successful reproduction of the species in the unpredictable cold desert.

Seed heteromorphism commonly exists in many plant species, such as members in Asteraceae, Poaceae, Brassicaceae, Chenopodiaceae (Venable, 1985; Sun et al., 2009; Yao et al., 2010). Morphologically heteromorphic fruits or seeds based on different color, size or presence/absence of WP have been widely investigated, e.g., *S. affinis*, *S. brachiata*, *S. korshinskyi*, *Suaeda aralocaspica* (Wang and Wei, 2007; Wei et al., 2007, 2008), in which different dormancy degree and germination ability are observed. In the present study, three types of seed were identified from different parts of *S. ferganica* plant, which differed not only in WP size, but in seed mass and germination or dormancy behavior, it is consistent with the previously reported data (Imbert, 2002). Theoretically, morphological features of seed usually reflect the aspects in ecological adaptation to maternal environments (Dey et al., 2016). Dispersal ability is one property of adaptation to harsh and variable environments (Mandák, 1997; Imbert, 2002), to which the WP size is an important feature. Long wing would be beneficial to escape the harshness away from their original habitats, which ensures seedling development in a suitable place and at the right time (Bhatt et al., 2016). It has been found that one seed morph has higher dispersal ability usually with little or no dormancy, while the other one has lower (or no) dispersal ability with higher dormancy (Lu et al., 2012; Baskin et al., 2014). Our experiments also indicate that larger size of WP of *S. ferganica* seed which had higher germination percentage could be dispersed to a farther distance from the mother plant. Moreover, the proportion of the farther dispersal units also has important significance in population expansion (Jansen et al., 2008). In the present study, the fruit (seed)-setting pattern of *S. ferganica* indicate that seeds with larger size of WP (farther dispersal unit) accounted for larger proportion of the total, it is consistent with the report on *S. affinis* (Wang and Wei, 2007; Wei et al., 2008). Our data and others suggest that certain combination of seed heteromorphism and seed-setting as well as dispersal pattern may be a unique adaptation strategy for some plant species like *S. ferganica* (in the present study) in the natural habitat.

The evolution in survival and fitness of plants to unpredictable or stressful environments has led to the development of various morphological and physiological adaptations on seed dormancy (Harper et al., 1970). Based on the responses of morphological and/or physiological aspects in dormancy breaking experiments, different dormancy types were defined by previous work of Baskin and Baskin (2014). In the present study, fresh LSs could germinate to a higher percentage (60–80%), that of the middle or the SSs was significantly lower than the LSs, the germination percentage of three types of seed all could be improved by stratification (breaking the constraint of WP) and GA_3_ treatment (improving the physiological activities), which suggests that *S. ferganica* could be the morphological and non-deep physiological dormancy type according to the previous classification (Baskin and Baskin, 2014), this was supported by other similar reports (Cadman et al., 2006; Wang et al., 2008). Before response to stratification and GA_3_ treatments, heteromorphic seeds of *S. ferganica* were much sensitive to light (white or different wavelength) and which was the dominant factor to improve germination in the present study. It has been reported that light requirement is usually associated with germination time regulation to some halophyte (Wang et al., 2008), which may protect seedling from environmental extremes (Finch-Savage and Footitt, 2017; Gul et al., 2013). In some light-dependent species, light alone can control and applies major effect on germination (Wei et al., 2008; Gul et al., 2013). In the present study, a significant difference in germination percentage existed between light and dark among three heteromorphic seeds of *S. ferganica*, which is in agreement with the response of *Sporobolus ioclados* (Khan and Gulzar, 2003), *Halostachys caspica* (Tobe et al., 2000) and *Sarcocornia perennis* (Redondo et al., 2004) to light. In addition, some species respond differently to various wavelength of light in germination (Borthwick et al., 1952). In the present study, germination percentage of heteromorphic seeds of *S. ferganica* was higher under yellow or red light than that of green or blue light and dark, which is similar to *Cattleya walkeriana*, *Lepidium virginicum*, and *L. densiflorum* (Toole et al., 1955; Islam et al., 1999) under different qualities of light. Taken together, seed dormancy of *S. ferganica* could be defined as light-sensitive combined with the morphological and non-deep physiological type.

It has been found that the accessory parts of seed usually apply significant effects on dormancy, e.g., seed coat, winged pericarp, bracteole, etc., which may physically or chemically regulate seed dormancy with the probable ions, chemicals or hormones in seed cover (Baskin et al., 2014; Bhatt et al., 2016). A certain amount of ions in seed bracteole of the genus of *Atriplex* (Koller, 1957; Khan and Ungar, 1985; Mandák and Pyšek, 2005) and the presence of abscisic acid in WP in *S. komarovii* (Takeno and Yamaguchi, 1991) have been detected and proven to be an important factor for seed dormancy. In the present study, compared to seed with WP, removal of WP could significantly increase germination percentage, which suggests that the WP itself or some substances in it might constrain seed germination. However, the ion concentrations (K^+^, Na^+^, Ca^2+^, Mg^2+^) in WP of *S. ferganica* seed (about 1.8 mM of total ions from 25 WP in 7 mL H_2_O in a 9 cm Petri dish) were not high enough to significantly inhibit germination [compared to the salt treatment (**Figure 11**) in our experiment]. Moreover, in the natural habitat of *S. ferganica*, large amount of melting snow water and the rainfall in spring may wash away some of the ions in WP. All these mean that ions in WP may not be the inhibitor for germination. Meantime, two phytohormones-GA_3_ and ABA were analyzed in the present study, which were not detectable at 10^-3^ mg⋅L^-1^ level of standard hormone (50 WP in 10 mL H_2_O). In combination with our exogenous applying of GA_3_ in germination experiment (**Figure 9**) and the results in *Suaeda salsa* that at least 10 μM ABA or 1 μM GA_3_ could affect seed germination (Li et al., 2015), suggesting that the level of GA_3_ or ABA in WP of *S. ferganica* seed should not apply significant effect on seed dormancy or germination. Taken together, our results suggest that the WP of *S. ferganica* seed may be just a mechanical barrier rather than the chemical effect on germination or dormancy.

In the desert habitat, broad temperature variation exists between day and night, and which correspondingly reflects on temperature range of seed germination of many desert plants, but different species show diverse optimum germination temperature (Wang et al., 2008; Gul et al., 2013). Relatively lower night/day temperature variation (N/DTV) (5°C/15°C, 10°C/20°C, or 15°C/25°C in the present study) significantly promoted germination of heteromorphic seeds of *S. ferganica*; otherwise the higher N/DTV (20°C/30°C in the present study) decreased the germination. Such a response of seed germination to temperature in the present study is corresponding to the environmental conditions of the natural habitat of *S. ferganica* in spring (**Figure 1**), when the temperature rises to about 5–10°C, and the soil was wet due to melting snow, just like experience of CS, such an effect for dormancy breaking has been reported in many other desert plants undergoing cold winter (Huang et al., 2003; Wei et al., 2007; Qu et al., 2008). The variable daily temperature is generally benefit to germination of dormant seeds (Wang and Wei, 2007; Wei et al., 2008), moreover, the responses were different with the heteromorphic seeds of *S. ferganica* in the present study, it implies that *S. ferganica* can adapt to wider temperature range at germination stage. Generally, dry storage or after-ripening process is needed for many annual halophytes to finish the final seed maturation (El-Keblawy, 2013). From an ecological view, such process sometimes applies an effect of avoidance of unfavorable conditions on seed germination (Jayasuriya et al., 2012; El-Keblawy and Bhatt, 2015). Seed vigor and germination percentage are highly variable through a long-time storage and strongly associated with their mother plant situation (Brits et al., 2015; Jha et al., 2015). Previous report showed that seeds of *Haloxylon* species would keep higher germination percentage for only 10 months on average (Wei and Wang, 2006). In the present study, seeds of *S. ferganica* from different calendar years presented diverse quality, e.g., seeds of 2013 could keep higher germination percentage for at least 15 months while seeds of 2014 were only 12 months. Our results suggest that seed persistence depends not only on seed vigor but on other environmental conditions in maternal habitat (Gremer et al., 2016; Jaganathan, 2016).

It has long been noticed that halophyte seeds can tolerate high salt concentration by entering forced secondary dormancy, and recover soon once the conditions are improved (Gupta et al., 2014). In the present study, LSs of *S. ferganica* were much more salt-tolerant compared to middle and small ones, similar results for dimorphic seeds were reported for *Atriplex triangularis* (Khan and Ungar, 1984), *Arthrocnemum indicum* (Khan and Gul, 1998), and *Suaeda salsa* (Li et al., 2005). When ungerminated *S. ferganica* seeds undergone high salt concentration were transferred to distilled water in our experiment, the seed viability was quickly released, which is consistent with other halophytes in inland cold salt desert (Zia and Khan, 2004; Wei et al., 2008). *S. ferganica*, distributes in an area with strong evaporation in spring season, which usually leads to accumulation of large amount of salts on the surface of the soil (**Table 1**) and consequently causes serious salinity stress in seed germination. However, spring rainfall can relieve such constraint by washing off the soil surface salts to improve the germination environments (**Figure 1**). Our data suggest that the heteromorphic seeds of *S. ferganica* present different responses to salt stress and can be forced into secondary dormancy to avoid high salinity, which should be a smart strategy to adapt the harsh natural habitats in the cold salt desert.

Lines of evidence indicate that weather conditions can be the important reason for variation of seed heteromorphism (Dullinger et al., 2004; Wei et al., 2008). The previous work showed that the proportion of larger seed might increase in response to tough environment in halophyte *Suaeda salsa* (Li et al., 2005; Wang et al., 2017) and *A. triangularis* (Khan and Ungar, 1984; Ungar, 1987). In the present study, the LS proportion of *S. ferganica* decreases in 2013 while increased in 2015; On the contrary, the SS proportion increased in 2013 but decreased in 2015. Based on analysis of the meteorological data of past 8 years (2008–2015) in the natural habitats of *S. ferganica* (**Figure 1**), we found that more precipitation at early spring and less at autumn (e.g., 2013) resulted in the lower proportion of LS but higher proportion of SSs, and seeds were more persistent; whereas less precipitation at early spring and more at autumn (e.g., 2015) led to higher proportion of LS but lower proportion of SS, and seeds were short-lived. It has been reported that germination time and seasonal climate changes (e.g., temperature, precipitation) can apply effects on seed morph ratio of offspring of *Suaeda corniculata* heteromorphic seeds in the original habitats (Yang et al., 2015). From this point, we speculate that difference in the environmental conditions in the natural habitat (e.g., temperature, precipitation), especially the precipitation in spring (from February to May) and in autumn (from September to October) might be responsible for the variations of seed heteromorphic ratio of *S. ferganica* and seed persistence in seed bank.

## Conclusion

**FIGURE 13:**
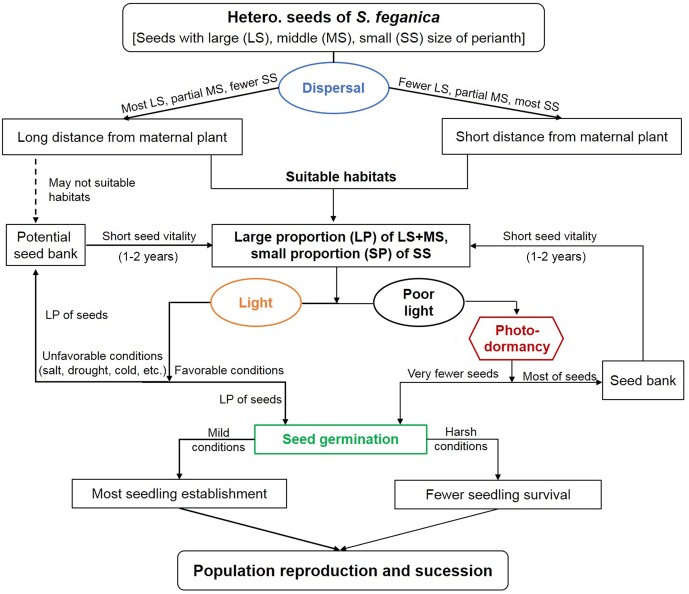
Proposed model of dynamics of reproduction for heteromorphic seeds of *S. ferganica* under various environmental conditions.

It has been reported that both seed polymorphism and seed bank can ensure enough amount of seedling establishment in unpredictable habitats and consequently promote population propagation (Thompson, 1981; Allessio Leck and Simpson, 1987; Baskin and Baskin, 1998), this seems to be the case with *S. ferganica* in the present study. Based on our results and other related viewpoint, we proposed a model for the cycle of heteromorphic seeds of *S. ferganica* in the cold desert as shown in **Figure 13**. There were three types of seeds (LS, MS, and SS) of *S. ferganica* according to the size of WP, which had different properties in dispersal ability and germinability. In suitable habitats, the mother plant can produce large or moderate proportion (LP or MP) of MS or LS, and small proportion (SP) of SS. As a photo-sensitive species, LP seeds of *S. ferganica* are expected to enter the potential seed bank under poor light and unfavorable conditions; otherwise, LP of seeds would immediately germinate to ensure large amount of seedling establishment and final population reproduction under light and favorable conditions. Therefore, it is likely that seed heteromorphism allows *S. ferganica* to gain multiple competitive advantages in unpredictable environments, and seed bank may control the best time for seed germination and seedling establishment, both of them can reduce the risks of spatial and temporal changes of habitats on seed germination, seedling establishment and population reproduction, which permits this species thriving in the harsh cold desert.

## Author Contributions

HL, YM, and JW design the experiments and methodology. YM, JW, and HL wrote the manuscript. YM and JZ conducted the experiments and collected the data. YM, JW, SZ, and YL analyzed the data. All authors contributed critically to the drafts and gave final approval for publication.

## Conflict of Interest Statement

The authors declare that the research was conducted in the absence of any commercial or financial relationships that could be construed as a potential conflict of interest.

## Footnotes

1 www.weather.com.cn
